# Evaluating the Microsatellite Instability of Colorectal Cancer Based on Multimodal Deep Learning Integrating Histopathological and Molecular Data

**DOI:** 10.3389/fonc.2022.925079

**Published:** 2022-07-05

**Authors:** Wenjing Qiu, Jiasheng Yang, Bing Wang, Min Yang, Geng Tian, Peizhen Wang, Jialiang Yang

**Affiliations:** ^1^ School of Electrical and Information Engineering, Anhui University of Technology, Maanshan, China; ^2^ Science System Department, Geneis Beijing Co., Ltd., Beijing, China; ^3^ Qingdao Genesis Institute of Big Data Mining and Precision Medicine, Qingdao, China

**Keywords:** microsatellite instability, H&E images, multi-omics data, multimodal deep learning, compact bilinear pooling

## Abstract

Microsatellite instability (MSI), an important biomarker for immunotherapy and the diagnosis of Lynch syndrome, refers to the change of microsatellite (MS) sequence length caused by insertion or deletion during DNA replication. However, traditional wet-lab experiment-based MSI detection is time-consuming and relies on experimental conditions. In addition, a comprehensive study on the associations between MSI status and various molecules like mRNA and miRNA has not been performed. In this study, we first studied the association between MSI status and several molecules including mRNA, miRNA, lncRNA, DNA methylation, and copy number variation (CNV) using colorectal cancer data from The Cancer Genome Atlas (TCGA). Then, we developed a novel deep learning framework to predict MSI status based solely on hematoxylin and eosin (H&E) staining images, and combined the H&E image with the above-mentioned molecules by multimodal compact bilinear pooling. Our results showed that there were significant differences in mRNA, miRNA, and lncRNA between the high microsatellite instability (MSI-H) patient group and the low microsatellite instability or microsatellite stability (MSI-L/MSS) patient group. By using the H&E image alone, one can predict MSI status with an acceptable prediction area under the curve (AUC) of 0.809 in 5-fold cross-validation. The fusion models integrating H&E image with a single type of molecule have higher prediction accuracies than that using H&E image alone, with the highest AUC of 0.952 achieved when combining H&E image with DNA methylation data. However, prediction accuracy will decrease when combining H&E image with all types of molecular data. In conclusion, combining H&E image with deep learning can predict the MSI status of colorectal cancer, the accuracy of which can further be improved by integrating appropriate molecular data. This study may have clinical significance in practice.

## 1 Introduction

Colorectal cancer (CRC) is a common digestive tract malignancy. CRC is the third largest cancer in the world, and the second leading cause of cancer-related death; the incidence rate and mortality rate of CRC were third and fifth, respectively, among all cancers in China, with more than 250,000 new patients and 140,000 deaths annually (1–3). Sporadic colorectal cancer (SCRC) accounts for about 85%, and hereditary nonpolyposis colorectal cancer (HNPCC) accounts for about 10%–15% of all CRC patients (4). SCRC is mainly affected by environment, diet, living habits, and chronic inflammation, which leads to the mutations of the “administrator gene” and “guard gene”; the mutations disrupt the mechanisms for inhibiting cell growth, promoting cell death, and maintaining cell stability. Among them, microsatellite instability (MSI) is involved in the occurrence of SCRC, with an incidence of 12%–15% (5). The value of MSI in the diagnosis, treatment response, and prognosis of CRC has attracted global attention (6–8).

MSI refers to the change in the length of normal microsatellites caused by the deletion or insertion of repeated bases compared with normal tissue cells (9). In 2001, Fukushima and Takenoshita (10) found that MSI significantly increased the random mutation rate of genes, especially the mutation of tumor-related genes, which is an important mechanism of tumorigenesis.

There is some evidence to support the use of pre-diagnostic MSI in clinical decision-making. First, MSI detection is recommended for the diagnosis of Lynch syndrome. Lynch syndrome is the most common hereditary colon cancer syndrome, which is associated with germline mutations in the MMR gene (MLH1, MSH2, MSH6, or PMS2) (11). MSI status helps to identify families with the syndrome. Second, MSI is one of the key factors affecting the prognosis of CRC, especially in early cases (12, 13). In general, patients with stage II CRC with high MSI (MSI-H)/MMR deficiency (d MMR) have a better prognosis than patients with microsatellite stability (MSS) and low MSI (MSI-L)/MMR (p MMR) (13). Third, MSI status can be used to evaluate therapeutic response, including fluoropyrimidine-based chemotherapy (14) and immunotherapy (15). Fluoropyrimidine (5-FU or capecitabine) is the pillar of the CRC chemotherapy strategy. It plays an important role not only in neoadjuvant therapy but also in prognosis treatment (16, 17). However, patients with MSI-H status are usually resistant to 5-FU-based chemotherapy (18). Immunotherapy is an emerging and promising treatment for CRC because MSI-H tumors have a large number of mutant neoantigens, which makes them sensitive to immune checkpoint inhibitors (19). Therefore, MSI status is crucial for selecting CRC treatment and evaluating the response to treatment (20).

In recent years, the deep learning method has become a newly developing method, which has shown excellent performance in the fields of computer vision (21, 22), speech recognition (23), and bioinformatics (24–27). Deep learning technology has the characteristics of end-to-end training, and can also represent abstract concepts or patterns level by level through deep neural networks (28). At the same time, researchers use the technology of transfer learning to transfer the network model pre-trained by Image Net to the classification task of pathological image segmentation by fine-tuning the classifier layer of convolutional neural network. In the 2016 CAMELYON breast cancer lymph node metastasis challenge, 25 of the 32 algorithms submitted by the contestants used convolution neural networks (CNNs) (29) including VGG-16 (30), GoogLeNet (31), and other well-known models such as (32). Xu et al. used pre-trained AlexNet to extract the features of brain tumor pathological image blocks and achieved 97.5% classification accuracy on the small-sample MICCAI 2014 brain tumor digital pathology challenge dataset. Yang et al. proposed a multimodal deep learning method to predict the recurrence and metastasis risk of Her2-positive breast cancer by integrating pathological image with clinical information (33). Ye et al. developed a deep convolution network to evaluate prognosis of cervical cancer (34). Ke et al. (35) used the knowledge distillation model of multistage CNN to classify MSI-H and MSS, and obtained an AUC = 0.802; Kather et al. (36) used ResNet18 to predict the histopathological sections of CRC, and the AUC obtained by MSI was 0.84.

With the increasing availability of high-throughput genomic and transcriptional data, there are several molecular biomarkers in The Cancer Genome Atlas (TCGA), including somatic mutation, copy number variation, gene expression, microRNA expression, and DNA methylation, which were used to track cancer (37–39) and predict cancer recurrence and metastasis (40). Hayes identified relevant microRNA and mRNA features that predict high-risk and low-risk patients with glioblastoma (GBM). Sun et al. integrated gene expression profile, CNA spectrum, and clinical data to predict the prognosis of breast cancer, achieving a good performance of AUC = 0.843.

Based on the feasibility of cancer prediction and multimodal fusion from the pathological image level, our goal was to compare these unimodal data and combinations to predict the MSI ability of CRC in a unified context and to explore whether multimodal data fusion can significantly improve prediction accuracy compared with single-mode data.

## 2 Materials and Methods

### 2.1 Data Description

We overlapped the H&E images data and omics data to obtain 353 sample sizes, of which 63 were labeled MSI-Hs, which were marked as 1; 290 cases were labeled MSSs, which were labeled as 0.

**Pathological image.** We used the method of Kather et al. to publish the CRC with hematoxylin and eosin stabilized (CRC-HE) dataset, including 100,000 pieces of 224 × 224 pixel H&E-stained pathological images that were divided into blocks; each pixel in the block corresponds to 0.5 μm × 0.5 μm organization. To eliminate the color difference of slices from different data sources in the process of production and scanning, all H&E images have been dyed and standardized according to the method of Macenko et al. (41).

**Multi-omics data.** Multi-omics data of CRC were downloaded from the TCGA database, including messenger RNA (mRNA), microRNA expression (miRNA), long non-coding RNA (lncRNA), DNA methylation (Met), and gene copy number variation (CNV). Their forms include Counts and FPKM. The difference between FPKM and Counts is that Counts is the original expression quantity that is not processed in the data background, although FPKM and Counts are data processing methods. In the analysis of this paper, the difference analysis part adopts the form of Counts, and the modeling analysis part adopts the form of FPKM. **Table 1** shows the characteristic dimensions of each omics data.

**Table 1:** The properties of the dataset.

Data Category	Abbreviation	Number of features
Messenger RNA	mRNA	19,531
MicroRNAs	miRNA	1,881
Long non-coding RNA	lncRNA	7,308
DNA methylation	Met	27,578
Copy number variation	CNV	60,483

### 2.2 Feature Extraction

#### 2.2.1 H&E Image Feature Representation Based on ResNet34

CNN is the latest algorithm for image recognition and classification because of its stable learning performance (42). CNN includes an input layer, a middle hidden layer, and an output layer. The middle-hidden layer is composed of multiple convolution layers, pooling layers, and full connection layers. CNN can be optimized through error backpropagation and gradient descent algorithm. However, after reaching a certain depth, increasing the number of layers of CNN cannot further improve the classification performance. Due to the vanishing gradient problem, the network convergence speed is slow and the classification accuracy is negative. ResNet is used to solve this problem. The difference between residual network and ordinary network is that jump connection is introduced, which can help the information of the previous residual block enter the next block stream unimpeded, improve the information flow, and avoid the problem of vanishing gradient and the degradation caused by the great depth of the network.

**Figure 1:**
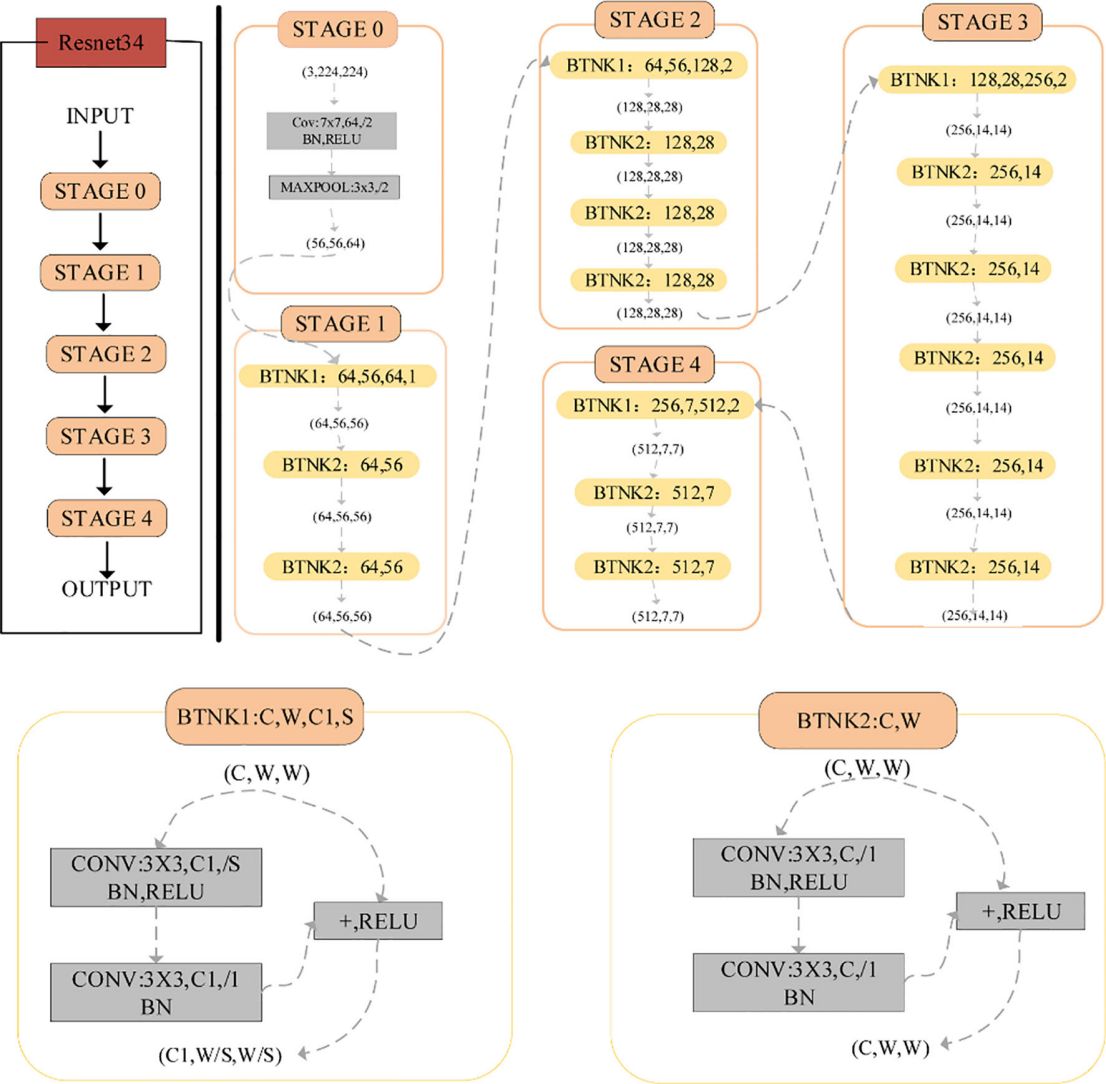
The network architecture of ResNet34.

ResNet is a large-scale CNN constructed from residual blocks. We used ResNet34 (**Figure 1**) to extract H&E image features. The architecture of ResNet34 is divided into four stages. Every Resnet architecture performed the initial convolution and max-pooling using 7 x 7 and 3 x 3 kernel sizes, respectively. The residual structure of BTNK1 can reduce the dimension, and the dimension is reduced by a 1 x 1 convolution kernel on the shortcut branch. It is worth noting that in Stage 2, Stage 3, and Stage 4, it is executed with stride 2; therefore, the size of the input will be halved in height and width, but the channel width will be doubled. When the image advances from one stage to another, the channel width will be doubled and the input size will be reduced by half. Finally, the network has an average pool layer, followed by a full connection layer containing 1,000 neurons.

#### 2.2.2 Feature Extraction of Multi-Omics Data

A common problem with high-throughput sequencing datasets is the so-called “Curse of dimensionality” (40). Variable selection is very important for interpretation and prediction, especially for high-dimensional datasets. In this work, we used the characteristic importance attribute of Random forest (Gini-index) (43) to deal with high-dimensional variables in omics data. Features with Gini-index greater than or equal to 0.005 were the most important features. Then, the multimodal data are simply spliced from the important features obtained from the single group data. Then, select according to the feature importance of random forest, and the feature with a Gini-index greater than 0.005 is regarded as the most important feature.

### 2.3 Feature Fusion

The most common fusion methods are concatenation, element-wise product, and element-wise sum. These simple operations are not as effective as the outer product, and complex relationships can be established between the two modes. However, the complexity of outer product calculation is too high. The *n*-dimensional vector calculated the outer product to obtain the *n* ^2^-dimensional vector. In this work, our fusion method was the multimodal compact bilinear (MCB) model. MCB maps the result of the outer product to low-dimensional space without explicit calculation of the outer product.

### 2.4 Screening of Differentially Expressed Genes

The R package “Deseq2” was used to identify differentially expressed genes (DEGs) in mRNA, miRNA, and lncRNA gene expression profiles. Genes with an adjusted *p*-value < 0.1 and a log2foldchange (LFC) > 0 were classified as upregulated genes, whereas those with an adjusted *p*-value < 0.1 and an LFC < 0 were classified as downregulated genes. Taking |log2 (foldchange)| ≥ 1 and the corrected *p*-value < 0.05 as the threshold, the genes with significant differences were selected. The R-Pack “heat map” shows significantly different genes. The R-Pack “cluster analyzer” is used for Gene Ontology (GO) enrichment analysis and calculation. R-Pack ggplot2 is used to generate enrichment pathways in significantly different genes.

### 2.5 Evaluation Metrics

Fivefold cross validation (5-f cv) is used to evaluate the accuracy of the algorithm.5-k cv: Divide the dataset into five equally, and take turns using four of them as training data and one as test data. The performance of the classification algorithm is estimated by averaging 5 test sets. For binary classification, the area under the subject operating characteristic curve (AUC), Accuracy (Acc), Precision, Recall, and F1_score are used to evaluate the performance of the model.

## 3 Results

### 3.1 The Overall Framework of This Study

**Figure 2:**
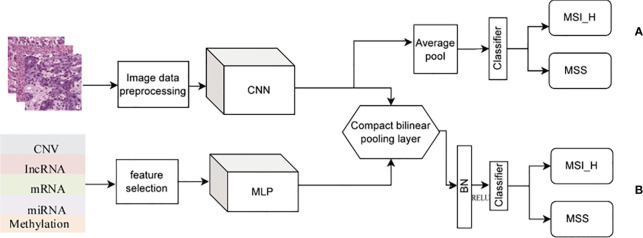
Experimental flowchart. **(A)** Only H&E images data. **(B)** H&E images combined with multi-omics data.

In this work, we studied the data in two parts. In the first part, the differences of mRNA, miRNA, and lncRNA were analyzed. In the second part, in the modeling analysis, we conducted two experiments (**Figure 2**). First, only the H&E image data were used to build the model and predict the classification (**Figure 2A**). Second, the H&E image was combined with omics for prediction and classification (**Figure 2B**), including H&E image combined with single omics data and H&E images combined with multi-omics data.

### 3.2 mRNA, lncRNA, and miRNAs Differ Significantly Between MSI-H and MSI-L/MSS Groups

**Figure 3:**
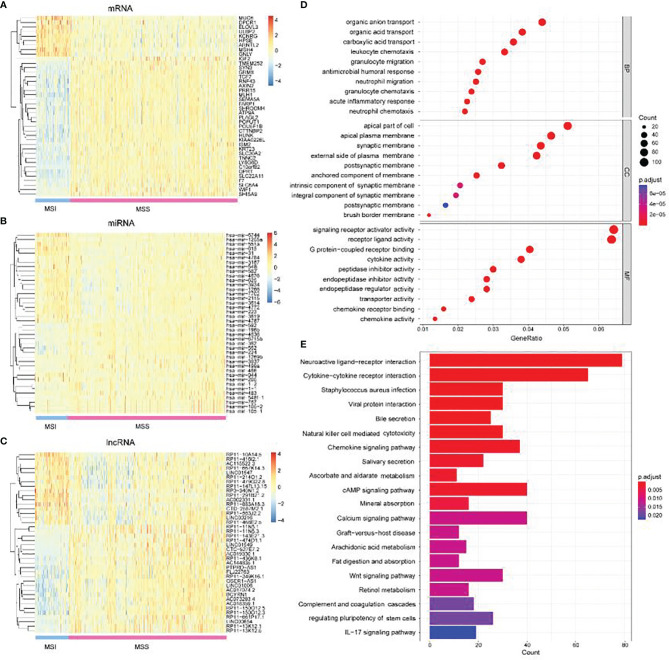
Differential analysis of mRNA, miRNA, and lncRNA. **(A)** Heat map of the top 40 differentially expressed genes of mRNA. **(B)** Heat map of the top 40 differentially expressed genes of miRNA and **(C)** lncRNA. **(D)** GO analysis, including BP, CC, and MF. **(E)** KEGG enrichment analysis.

We comprehensively analyzed the differential expression of mRNA, lncRNA, and miRNA between MSI-L/MSI-H and MSS groups. In the lncRNA group, we obtained 1,130 upregulated expressions and 631 downregulated expressions. A total of 172 upregulated expressions and 125 downregulated expressions were obtained in miRNA. In the mRNA group, 5,210 upregulated genes and 5,466 downregulated genes were obtained. After strictly restricting the adjusted *p*-value, we obtained 663 significantly differentially expressed lncRNAs, 61 significantly differentially expressed miRNAs, and 1,898 significantly different mRNA genes (see **Supplementary Tables 1–3**). As shown in **Figures 3A–C**, we used the first 40 significant difference expressions to draw the heat map.

GO analysis was used to annotate the function of DEGs between MSI-H and MSI-L/MSS. In the biological process (BP) category, genes with significant differences were mainly enriched in organic acid, organic anion, and carboxylic acid transport. For cell component (CC) categories, genes with significant differences were mainly clustered in the apical part of the cell. In the binding molecular function (MF), significantly different genes were mainly involved in signaling receptor activator activity and receptor–ligand activity (**Figure 3D**). Further KEGG enrichment analysis was carried out to explore the potential pathological pathway of cancer. As shown in **Figure 3E**, the first two significant enrichment pathways were neuroactive ligand–receptor interaction and cytokine receptor interaction. Our significantly different genes were involved in these pathways, which may also contribute to the diagnosis of cancer. For example, the *MUC6* gene is one of the mucin genes that make up the gastric mucosa, and its expression is downregulated in precancerous lesions and gastric cancer tissues (44). Dpcr1 *DPCR1*(Mucl3*MUCL3*) is a protein-coding gene. *MUCL3* may regulate NF kappa B signaling and play a role in cell growth.

### 3.3 H&E Images Combined With DNA Methylation Performed Best in Predicting MSI of Colorectal Cancer

**Figure 4:**
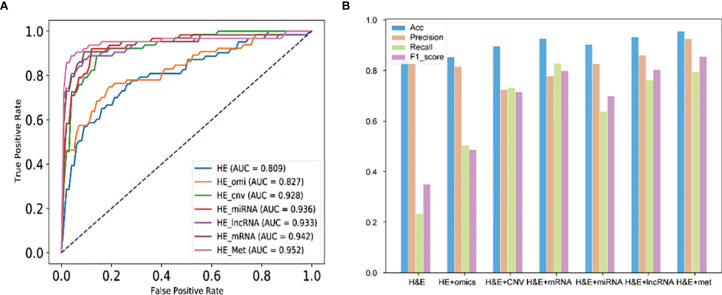
Performance of H&E images and images combined with omics data. **(A)** The AUC score of image and image combined with omics data. **(B)** Performance of each mode in Accuracy, Precision, Recall, and F1_score index. HE_omi: H&E image features combined with multi-omics features.

We evaluated the performance of images combined with omics data in predicting the MSI of CRC. 5-f cv was used to train ResNet34. As shown in **Figure 4A**, the prediction result of H&E images combined with DNA methylation (ROC = 0.952) was higher than that of H&E images, H&E images combined with multi-omics, and image combined with other omics data. In addition to H&E images combined with methylation, H&E images combined with other omics was lower than the prediction result of image in precision index. In Acc, Recall, and F1_ score index, the prediction results of image combined with omics were higher than those of image (**Figure 4B**).

## 4 Discussion

As we all know, MSI is widely considered as an indicator of prediction and prognosis. It has been well studied in several types of human cancers. In CRC, about 15% to 20% of CRC cases are found to be associated with MSI-H. Therefore, MSI states that detection is particularly important for CRC and is recommended by current clinical guidelines (6, 45). With the continuous development of computer deep learning technology, computer-aided diagnosis and prognosis prediction based on H&E staining images has attracted more and more attention because of its advantages of high speed, low cost, and no trauma. Multimodal fusion is a typical interdisciplinary field and has gradually become a research hotspot. In many studies, some results have been achieved (46–48). In conclusion, the accuracy of the image-based prognosis prediction model needs to be further improved.

In this study, we systematically analyzed the differences in mRNA, lncRNA, and miRNA omics data between MSI-H and MSI-L/MSS groups, and compared the classification performance of image and image data combined with omics data to predict the MSI of CRC. In this experiment, by comparing the results of ROC, we found that H&E image combined with Met had the best performance in predicting the MSI of CRC. The result of H&E image combined with all omics data was lower than that of image combined with single omics data and higher than that of H&E images.

Our study has some limitations. First, the selected omics data were the cancer sample construction and evaluation model, not the adjacent data. Only the differences between MSI-L/MSS and MSI-H in cancer samples were studied. Second, we do not have independent datasets for validation, because we cannot find other databases to provide the required data except for the TCGA database. Finally, our multi-omics feature was just simple splicing of different single omics. It is best to test the effects of interactions between omics because the genes of each omics are not completely independent. Therefore, in our follow-up study, we will try to include para-cancerous samples, including independent test samples, and add interactive items and new classification models to improve the prediction accuracy.

## 5 Conclusion

To sum up, we integrated molecular biological information and images to classify and predict the MSI of CRC. This is the first study to compare the ability of different modes in predicting the MSI of CRC under the same conditions, including the same dataset, the same preprocessing scheme, and the same classification algorithm. There were significant differences in mRNA, lncRNA, and miRNA omics data between MSI-H and MSI-L/MSS groups. By comparing the results of ROC, we found that H&E images combined with Met had the best performance in predicting the MSI of CRC. The result of image combined with all omics data was lower than that of image combined with single omics data and higher than that of H&E images.

## Data Availability Statement

Publicly available datasets were analyzed in this study. These data can be found here: https://www.cancer.gov/about-nci/organization/ccg/research/structural-genomics/.

## Author Contributions

JLY and PW designed the study. WQ, JSY, BW, MY, and GT performed the study, analyzed the data, and interpreted data. WQ and JLY wrote the manuscript. JSY, BW, MY, GT, and PW reviewed the manuscript. All authors contributed to the article and approved the submitted version.

## Funding

This research was funded by the National Natural Science Foundation of China (numbers 51574004 and 62172004), the Natural Science Foundation of the Higher Education Institutions of Anhui Province, China (KJ2019A0085), the Academic Foundation for Top Talents of the Higher Education Institutions of Anhui Province (gxbjZD2016041), and the Educational Commission of Anhui Province (KJ2019ZD05).

## Conflict of Interest

Authors WQ, JLY, GT, and MY were employed by Geneis Beijing Co., Ltd., Beijing.

The remaining authors declare that the research was conducted in the absence of any commercial or financial relationships that could be construed as a potential conflict of interest.

## Publisher’s Note

All claims expressed in this article are solely those of the authors and do not necessarily represent those of their affiliated organizations, or those of the publisher, the editors and the reviewers. Any product that may be evaluated in this article, or claim that may be made by its manufacturer, is not guaranteed or endorsed by the publisher.
